# Factors associated with compliance with the recommended frequency of postnatal care services in three rural districts of Tanzania

**DOI:** 10.1186/s12884-015-0769-8

**Published:** 2015-12-21

**Authors:** Almamy M. Kanté, Christine E. Chung, Anna M. Larsen, Amon Exavery, Kassimu Tani, James F. Phillips

**Affiliations:** Mailman School of Public Health, Columbia University, 60 Haven Avenue, New York, 10032 USA; Ifakara Health Institute, PO Box 78373, Mikocheni, Dar es Salaam, Tanzania; Swiss Tropical and Public Health Institute, Socinstrasse 57, CH-4002 Basel, Switzerland; University of Basel, Petersplatz 1, CH-4003 Basel, Switzerland

**Keywords:** Postnatal care, Compliance with the recommended frequency, Ifakara, Rufiji, Tanzania

## Abstract

**Background:**

High neonatal mortality persists in Tanzania. Rates of decline are slow, in part because postnatal care (PNC) services for addressing this problem remain severely underutilized. This study assesses factors associated with utilization of PNC among mothers in rural Tanzania.

**Methods:**

This study analyzed household survey data collected in 2011 to understand health service utilization patterns among women of reproductive age and children less than 5 years of age in the Rufiji, Kilombero, and Ulanga districts of Tanzania. A total of 889 mothers were eligible for the current analysis. Multinomial logistic regression was used to determine factors associated with the likelihood of mothers seeking the WHO recommended PNC visits.

**Results:**

The percent of newborns and their mothers with full PNC was low (10.4 %). Factors explaining PNC completion were district of residence, ethnic group, pregnancy wantedness, ANC attendance, place of delivery, and any incidence of newborn. Mothers of unwanted pregnancies were less likely to attend PNC services compared to mothers of wanted pregnancies [for at least two PNC: aRRR = 0.57, 95 % CI 0.35–0.94]. Sick newborns were more likely to receive PNC than newborns who were not sick during the first month after childbirth [for at least two PNC, aRRR = 3.52, 95 % CI 2.12–5.86]. Mothers who attended ANC services more frequently were more likely to receive PNC services compared to those who had attended fewer than 2 ANC services [for 1 PNC, aRRR = 1.89, 95 % CI 1.23–2.90]. Mothers who delivered at a health facility were less likely to attend PNC services compared to mothers who delivered outside a facility [for at least 2 PNC: aRRR = 0.42, 95 % CI 0.26–0.76]. Model with interactions between ANC attendance and place of delivery shown that only ANC attendance had a positive and statistically significant effect on PNC visit.

**Conclusion:**

To achieve the WHO recommended number of PNC in rural Tanzania, our findings suggest the need to provide PNC through the community-based primary health care. Efforts to improve coverage of PNC should include expanding health education and counseling during childbirth and neonatal period to more effectively advocate PNC for newborns perceived to be healthy.

## Background

Of the 130 million live births that occur worldwide annually, an estimated 3.7 to 4 million deaths occur in the first 28 days of life contributing to 40 % of the total deaths in children under 5 years of age [1, 2]. About half of these deaths occur on the first day of life, and more than two-thirds within the first week of life [3]. About 99 % of all neonatal deaths occur in Low- and Middle-Income Countries (LMIC), and about half also occur at home and thus are at risk of being uncounted [2, 4].

In response to this problem, the World Health Organization (WHO) promotes basic postnatal care (PNC). Specifically, WHO recommends that mothers and newborns receive initial PNC care within the first 24 h after delivery and a minimum of three additional PNC visits within 48–72 h, and 7–14 days, and 6 weeks after delivery [5]. Recommendations concerning number, timing, and content of PNC are based on clustering of neonatal deaths at certain ages, common causes of neonatal death, and the most impactful timing for delivering interventions. The first 6 weeks of life can be divided into three phases, each represented by associated risks and corresponding priorities for the provision of care to the newborn. Care received involves monitoring for danger signs in the infant’s breathing, temperature, breastfeeding, and movement, as well as counseling the mother about healthy nutrition and lifestyle practices. The *immediate postnatal period,* corresponding to the first 24 h after birth, is associated with the greatest concentration of fatal risks to the newborn. These include birth asphyxia, trauma, breathing issues related to preterm birth, and sepsis. The *early postnatal period,* corresponding to days 2 through 7 is a period when sepsis, malaria, and other infectious disease risks are prominent. The *late postnatal period* extends from days 8 through 42 or weeks 2 through 6, when risks of childhood infections that are concentrated in week 1 remain as dominant causes of death, but at gradually diminishing levels of risk as days of age progress [6].

Research shows that achieving this recommended three clinical visit regimen of care at a 90 % level of coverage could avert up to 310,000 newborn deaths per year in Africa [7]. Evidence is not completely consistent, however. Some studies provide strong evidence of the link between PNC and neonatal survival [8, 9], while others have found no or weak associations [10, 11]. Particular PNC promoted practices for preterm deliveries, such as ‘*kangaroo care*’ reduce neonatal mortality when applied [7, 12–14], but implementation of this practice is often incomplete and suboptimal. Despite considerable evidence that the life-saving potential of PNC could be more effectively marshaled if implementation challenges are addressed, this component of primary health care has largely been overshadowed by the attention given to antenatal care (ANC) and promotion of the provision of skilled attendance at birth [10].

While there is mounting global investment in improving neonatal survival, there are often gaps in both the coverage and the quality of PNC services in much of the developing world. Often when PNC services are available, they are not comprehensive [15]. Most of studies also focused on the determinants of at least one component of PNC. Yet, WHO recommends at least three components of PNC [6].

In Tanzania, the coverage of health interventions is lowest during childbirth and the postnatal period [16], which are the periods when such care is most essential [2, 17]. Studies examining utilization of health services and health practices in general within Tanzania during the postnatal period are scarce, with most of the focus on perinatal services addressed instead to post-partum family planning, ANC, and midwifery care [18–25]. Several studies in LMIC have identified factors that are associated with PNC exposure: maternal educational attainment [26–31], household relative socioeconomic status [29–35], parental occupations and employment status [26, 28], and geographic factors, such as household distance to health facilities [29–34, 36–38]. Despite such results, however, evidence regarding factors influencing health care seeking behavior for PNC is inconsistent. Chakraborty et al. (2002), for example, found no significant effect of education or distance on utilization of health services during the postnatal period [39].

Few studies have looked into the factors associated with the WHO recommended frequency of PNC. This study is among the first to determine the extent to which mothers with recent pregnancies received the WHO recommended PNC services at a health facility or at a community with a traditional birth attendance or a village health worker (i.e., service utilization rate). Additionally, the study aims to identify individual characteristics and contextual factors associated with utilization of PNC among mothers with recent pregnancies in the Rufiji, Kilombero, and Ulanga districts of Tanzania.

## Methods

### Study area and population

The paper presents results from the analysis of a cross-sectional household survey conducted in the Rufiji, Kilombero, and Ulanga districts in Tanzania from May-July 2011. This survey sought to assess health behaviors and service utilization patterns among women of reproductive age (15 to 49 years) and children less than 5 years of age [40]. The survey was to provide baseline estimates for the Connect Project, which is currently being implemented as a randomized cluster trial (50 villages intervention, villages receiving community health workers (CHWs), and 51 villages comparison, villages not receiving CHWs) in these three districts. The Connect Project tests the child mortality impact of posting CHWs for providing preventive, promotive, and curative antenatal, newborn, child, and reproductive health care [41].

Rufiji district is located in the Pwani region, while the Kilombero and Ulanga districts are both located in the Morogoro region. The survey was conducted in two existing Health and Demographic Surveillance System (HDSS) platforms of Ifakara (covering Kilombero and Ulanga districts) and Rufiji district. In 2011, the two HDSS covered a population of 365,000. Across the three districts, more than 95 % of women currently attend at least one ANC, about 65 % deliver at the health facility, mainly dispensaries, and 75 % of births are assisted by health professionals. Most of the population also receives care from traditional healers and traditional birth attendants. The neonatal mortality rate has stabilized around 25 deaths per 1000 live births in both Ifakara and Rufiji HDSS over the past decade [42, 43].

### Data analysis

The sample for this analysis included 889 mothers who had completed a pregnancy within the two years preceding the survey. For each mother, proportions were calculated regarding whether she attended at least three PNC, two PNC, one PNC or no PNC within the neonatal period. Multinomial logistic regression was used to calculate the measures of association (Relative Risk Ratios (RRR)) between each of the independent variables (mother and household characteristics) and the likelihood of mothers seeking PNC services. Differences were tested for significance at a 5 % significance level. Analyses were completed using the STATA 13.1.

Correlates of PNC seeking were selected based on the literature. Demographic characteristics of mothers were: age group (less than 20, between 20 and 34, and more than 35 years old), educational attainment (no education, primary and secondary or higher level of education), marital status (married, divorced, widowed, or single), religion (Muslim, Christian, or other), ethnic group, district of residency (Kilombero, Ulanga, and Rufiji), and place of residence (rural and semi-urban). Rufiji was used as the reference group for analysis because its geographic characteristics and socio-economic profile are distinct from those of Kilombero and Ulanga.

An index of household wealth, based on household assets such as source of drinking water, ownership of a toilet and type of toilet was created using principal component analysis (PCA) methods [44] and used to group households into wealth quintiles. Household and health facility-level data were linked to geo-tagged household and facility locations in ArcGIS version 10 [45]. Using the near function, the straight-line distances from the household of the mother to the point location of the nearest facility were measured in kilometers (less than 1 km, between 1 and 3 km, between 3 and 5 km, and more than 5 km) to analyze the extent to which distance to nearest health facility was a barrier to seeking health services during the postnatal period.

Other variables of interest were related to the characteristics of pregnancy: order (first pregnancy, between second and fifth, and sixth or more), wantedness (wanted: intended or mistimed, and unwanted), to mother behaviors during pregnancy (frequency of ANC: less than 3, 3 and 4 ANC or more, and timing of ANC: early, late and very late,); place of delivery (at the facility and outside the facility); and mother’s knowledge of health practices related to pregnancy and delivery, and to danger signs for newborns (high, moderate and low), and health status of the newborn.

### Ethical considerations

The Columbia University Medical Center Institutional Review Board (Protocol AAAF3452), the National Institute for Medical Research of the Medical Research Coordinating committee (NIMR-CC) (NIMR/HQ/R.8a/Vol.IX/1203) and the Ifakara Health Institute’s Institutional Review Board (IHI/IRB/No. 16-2010) have all approved this study. Participation in the survey was entirely voluntary and participants signed an informed consent form preceding the interview. For each participant younger than 18 years of age, consent was sought from her respective parent/guardian.

## Results

### Sample characteristics and utilization of postnatal care (PNC) services

The average age of the mothers was 27.8 ± 7.6 years. Twenty-five percent of mothers did not have any formal educational while 70.3 % attended the primary-level of education and only 5.1 % attended the secondary or higher-level. There were over 20 ethnic groups represented in the sample, the top four being the Ndengereko, Pogoro, Ngindo and Sukuma groups. Half of the mothers were Muslim while 42.6 % were Christian and 6.5 % were traditional or other beliefs. More than half of the mothers resided in the Kilombero district (58.5 %), 24.9 % living in Rufiji and 16.7 % living in Ulanga.

For one-fifth of mothers, the newborn recently delivered was their first-born. Less than half (47.0 %) of the newborns were from intended pregnancies, 33.6 % were mistimed while 15.1 % were from unwanted pregnancies. One-third of mothers were knowledgeable about general health practices related to pregnancy and could identify at least three danger signs regarding newborn health. About 18 % of the mothers reported that their newborn had at least one episode of illness in the first month after birth. Almost all mothers (99.8 %) attended at least one antenatal visit and only 43.0 % attended the WHO recommended four visits. Nearly three-quarters (74.5 %) of mothers delivered at the health facility.

Slightly more than one-fourth of the mothers (27.8 %) resided less than 1 km to the nearest health facility (dispensary, health center or hospital). Two-thirds lived nearest to a dispensary while one-third lived nearest to a health center or hospital.

Following the birth, mothers and their newborns received an average of 1.1 PNC at a facility or at home, ranging from 0 to 13 visits. Slightly more than one-third (34.5 %) of mothers and their newborns had not attended a PNC checkup while 43.5 % had attended once, 11.6 % had attended twice, and only 10.4 % had attended at least three PNC, as is recommended. Only 8.6 % of mothers and their newborns had received PNC visit at home from community health workers (traditional birth attendance or village health worker) (Table 1).

**Table 1:** Maternal, pregnancy, continuum of care and nearest health facility characteristics and postnatal care prevalence (*n* = 889)

Characteristic		n (%)
*Maternal socio-demographic*		
Age (years)	Mean age = 27.8; SD = 7.6; Range 15–51	
	15 years ≤ 19 years	132 (14.85)
	20 years ≤ 34 years	548 (61.64)
	≥35	209 (23.51)
Educational attainment	Secondary and plus	45 (5.06)
	Primary	625 (70.30)
	None	219 (24.63)
Marital status	Married	709 (79.75)
	Unmarried	180 (20.25)
Religion	Muslim	452 (50.84)
	Christian	379 (42.63)
	Traditional/Other	58 (6.52)
Ethnic Group	Ndengereko	137 (15.41)
	Pogoro	127 (14.29)
	Sukuma	102 (11.47)
	Ngindo	126 (14.17)
	Other	397 (44.66)
Wealth quintiles	First (Poor)	209 (23.51)
	Second	216 (24.30)
	Third (Middle)	151 (16.99)
	Fourth	136 (15.30)
	Fifth (Least poor)	99 (11.14)
	Non Response	78 (8.77)
District	Rufiji	221 (24.86)
	Kilombero	520 (58.49)
	Ulanga	148 (16.65)
Residence	Rural	721 (81.10)
	Urban/semi-urban	168 (18.90)
*Pregnancy and maternal and child health (MCH) knowledge*		
Parity	First pregnancy	181 (20.36)
	2–5 pregnancies	510 (57.37)
	6+ pregnancies	198 (22.27)
Wantedness of pregnancy	Intended	418 (47.02)
	Mistimed	299 (33.63)
	Unwanted	134 (15.07)
	Non response	38 (4.27)
*Mother’s knowledge of:*	Health practices related to pregnancy	
	Comprehensive	310 (34.87)
	Moderate	359 (40.38)
	Low/None	220 (24.75)
	Immediate breastfeeding after delivery	
	No	277 (31.16)
	Yes	612 (68.84)
	Danger signs of newborn health	
	3 or more	325 (36.56)
	2	310 (34.87)
	Less than 2	254 (28.57)
Mother’s decision-making about where to give birth	No	413 (46.46)
	Yes	476 (53.54)
Difficulties in getting postnatal care	No	808 (90.89)
	Yes	81 (9.11)
Illness of child during first month	No	730 (82.11)
	Yes	159 (17.89)
*Indicators related to the continuum of care*		
Family planning method use ever	No	568 (63.89)
	Yes	321 (36.11)
Antenatal care visits	Less than 3	140 (15.75)
	3	367 (41.28)
	4 or more	382 (42.97)
Antenatal care initiation	Very late (Third trimester)	89 (10.01)
	Late (Second trimester)	631 (70.98)
	Early (First trimester)	161 (18.11)
	Don’t know	8 (0.90)
Place of delivery	At health facility	662 (74.47)
	Not at health facility	227 (25.53)
*Nearest health facility*		
Ownership	Government	427 (48.03)
	Private	462 (51.97)
Type	Dispensary	671 (75.48)
	Health center or Hospital	218 (24.53)
Distance to health facility (km)	Mean (SD) = 3.139 (3.585)	
	<1 km	247 (27.78)
	1–2.99 km	336 (37.80)
	3–4.99 km	137 (15.41)
	> = 5 km	169 (19.01)
*Postnatal care prevalence*		
Postnatal care visits (facility or home)	Mean visit = 1.09; SD = 1.16; Range = 0–13	
	No	307 (34.53)
	1	387 (43.53)
	2	103 (11.59)
	3 or more	92 (10.35)

Complete compliance with the WHO recommended three visit regimen of PNC is uncommon in this context. For this reason, we combined the classes for two visits with three and over in the multinomial regression analyses.

### Determinants of postnatal care (PNC) services utilization

Mothers and newborns who lived in Kilombero and Ulanga were less likely to receive PNC compared to those who lived in Rufiji district in unadjusted bivariate analysis as well as multivariate analysis adjusted for other covariates [for at least 2 PNC, aRRR Kilombero = 0.46, 95 % CI 0.22–0.97; aRRR Ulanga = 0.12, 95 % CI 0.04–0.33]. Mothers of unwanted pregnancies were less likely to attend to PNC compared to mothers of unwanted pregnancies [for at least 2 PNC, aRRR = 0.57, 95 % CI 0.35–0.94]. Sick newborns were more likely to receive PNC than newborns who were not sick during the first month after childbirth [for at least 2 PNC, aRRR = 3.52, 95 % CI 2.12–5.86]. PNC utilization was also significantly associated with ethnic group.

Mothers who attended ANC services more frequently were more likely to receive PNC services compared to those who had attended fewer than 2 ANC services [for 1 PNC, aRRR = 1.89, 95 % CI 1.23–2.90; for at least 2 PNC, aRRR = 1.65, 95 % CI 0.96–2.82, *p* = 0.068]. Surprisingly, mothers who delivered at the facility were less likely to attend to PNC as compared to those who delivered outside the health facility [for 1 PNC, aRRR = 0.41; 95 % CI 0.28–0.61; for at least 2 PNC, aRRR = 0.42, 95 % CI 0.26–0.67] (Table 2).

**Table 2:** Adjusted relative risk ratio (aRRR) and 95 % confidence interval (CI) of maternal socio-demographic, pregnancy, continuum of care and nearest health facility characteristics on the likelihood to attend at postnatal care services (*n* = 889)

Variable	Category	1 VS No PNC	2+ VS No PNC
		(95 % CI)	(95 % CI)
*Maternal socio-demographic*			
Age (years)	15 ≤ 19 years	Ref.	Ref.
	20 ≤ 34 years	0.67 (0.38–1.16)	0.72 (0.37–1.39)
	≥35 years	0.52 (0.26–1.06)^+^	0.79 (0.34–1.84)
Educational attainment	Primary and plus	Ref.	Ref.
	None	0.90 (0.60–1.35)	0.76 (0.46–1.25)
Religion	Muslim	Ref.	Ref.
	Christian	0.90 (0.60–1.34)	1.10 (0.68–1.78)
	Traditional/Other	0.81 (0.32–2.04)	0.22 (0.05–1.00)^+^
Ethnic Group	Ndengereko	Ref.	Ref.
	Pogoro	1.94 (0.85–4.42)	2.82 (1.09–7.31)*
	Sukuma	1.48 (0.55–4.00)	3.07 (0.97–9.71)^+^
	Ngindo	1.90 (0.85–4.23)	2.40 (0.96–6.00)^+^
	Other	2.74 (1.35–5.56)**	2.34 (1.03–5.28)*
District	Rufiji	Ref.	
	Kilombero	0.53 (0.28–1.01)^+^	0.46 (0.22–0.97)*
	Ulanga	0.49 (0.23–1.04)^+^	0.12 (0.04–0.33)***
*Pregnancy and maternal and child health knowledge*			
Parity	First pregnancy	Ref.	Ref.
	2–5 pregnancies	1.02 (0.63–1.66)	0.82 (0.46–1.46)
	6+ pregnancies	1.22 (0.64–2.34)	0.75 (0.34–1.68)
Wantedness of pregnancy	Wanted	Ref.	Ref.
	Unwanted	0.57 (0.38–0.85)**	0.57 (0.35–0.94)*
*Mother’s knowledge of*	Health practices related to pregnancy		
	Comprehensive	Ref.	Ref.
	Moderate	0.73 (0.50–1.06)^+^	0.77 (0.49–1.20)
	Low/None	0.74 (0.48–1.14)	0.59 (0.34–1.04)^+^
	Danger signs of newborn health		
	High	Ref.	Ref.
	Moderate	1.31 (0.90–1.92)	1.14 (0.73–1.79)
	Low/None	1.05 (0.71–1.56)	0.66 (0.40–1.09)^+^
Illness of child during first month	No	Ref.	
	Yes	2.16 (1.35–3.45)**	3.52 (2.12–5.86)***
*Continuum of care indicators*			
Family planning method use ever	No	Ref.	Ref.
	Yes	1.18 (0.84–1.65)	0.96 (0.64–1.45)
Antenatal care visits	Less than 3	Ref.	Ref.
	3+	1.89 (1.23–2.90)**	1.65 (0.96–2.82)^+^
Place of delivery	Not at health facility	Ref.	Ref.
	At health facility	0.41 (0.28–0.61)***	0.42 (0.26–0.67)***

***p<0.001, **p<0.01, *p<0.05, ^+^p<0.10

Model with interaction terms between ANC attendance and place of delivery shown that the ANC attendance had a statistically significant effect on PNC visit. Using *lincom* command to test estimates for linear combinations, results did not show a statistically significant effect. Only the main effect has been interpreted here. When the delivery occurred outside a health facility, the RRR of a woman who attended three or more ANC having PNC services is higher that a woman who attended less than 3 ANC visits [for 1 PNC: aRRR = 2.81; 95 % CI 1.32–6.00; for at least 2PNC, aRRR = 4.50, 95 CI 1.55–13.11]. When ANC attendance is low (less than 3 visits), the effect of the place of delivery on PNC visit is not statistically significant (Table 3).

**Table 3:** Adjusted relative risk ratio (aRRR) and 95 % confidence interval (CI) of maternal socio-demographic, pregnancy, continuum of care and nearest health facility characteristics on the likelihood to attend at postnatal care services (model with interaction terms between “place of delivery and ANC visits” (*n* = 889)

Variable	Category	1 VS No PNC	2+ VS No PNC
		(95 % CI)	(95 % CI)
*Maternal socio-demographic*			
Antenatal care visits	Less than 3	Ref.	Ref.
	3+	2.81 (1.32–6.00)**	4.50 (1.55–13.11)**
Place of delivery	Not at health facility	Ref.	Ref.
	At health facility	0.62 (0.27–1.40)	1.31 (0.42–4.07)

Controlled for socio-demographic factors (age group, education attainment, religion, ethnic group, district of residence), factors related to pregnancy (parity, wantedness), mother’s knowledge of risk factors during pregnancy, delivery and the postnatal period, newborn health status and use of family planning services

Others models with interaction terms did not show significant association between “Age group and maternal education, “Illness of child during first month and place and delivery” or “maternal education and distance to the nearest facility

**p<0.01

PNC utilization was significantly associated (at a 10 % level of significance) with socio-demographic factors (age group and religion) and mother’s knowledge of health practices related to pregnancy and newborn health. Further associations were only statistically significant in the bivariate regression for socio-demographic factors (education attainment) and factors related to pregnancy (parity) and care seeking behavior along the continuum of care (use of family planning).

Such associations were not statistically significant even in the bivariate regression for socio-demographic factors (marital status, the wealth quintiles of the household and place of residence), the characteristics of pregnancy (timing for ANC), and the characteristics nearest health facility (distance, ownership and type).

## Discussion

The present study is among the first to determine maternal, newborn health, pregnancy, and household characteristic factors that are associated with compliance with the WHO recommended intensity of PNC. Just as Tanzania is predominantly rural (74 %), the focus of this study is a population of women from three rural districts where their the level of PNC utilization is related to predictors of care seeking for themselves and their newborns.

The rate of achieving at least one PNC visit among mothers and their newborns in our study area (65.5 %) was high relative to corresponding national estimates in rural areas (30.4 %) [16] and also high relative to levels observed in other sub-Saharan countries, such as Kenya 42.0 % [46], Malawi 50.0 % [47], Rwanda 19.2 % [48], Nigeria 34.1 % [28]. The high utilization rate for one visit found in this study may be an artifact of our decision to merge information about PNC received by the baby only, by the mother only, and by both the baby and the mother together, under the assumption that PNC services are benefiting both mothers and newborns. Nonetheless, the utilization rate for at least one PNC for mothers alone was 33 %, which is comparable to rural estimates in Tanzania.

Although three PNC service encounters are recommended within 6 weeks after childbirth, only 10.4 % of the deliveries were associated with this level of care. The low level of complete PNC compliance may arise from a combination of structural determinants, such as poor access to services, low coverage of PNC services at formal health facilities and perceived lack of services at such facilities by mothers, perceived lack of importance of seeking PNC services, a shortage of community providers making routine home visits, costs and transportation difficulties, and other cultural, geographic or financial barriers [15, 20, 49]. Global experts advise that in situations of poor utilization of formal health care systems, PNC should be provided by community health workers who are trained to provide such care during routine home visits [15, 49]. Our study found that only 8.4 % of mothers and their newborns received PNC visit at home from community health workers. The Connect Project’s trained cadre of community health workers can directly address this health need [41].

Regarding the issue of the factors associated with compliance with WHO recommended frequency of PNC services, mothers who declared that the pregnancy was unwanted, those who did not attended more frequently to ANC services or those who had low knowledge of risk factors during delivery and the postnatal period were less likely to utilize PNC services, results confirmed by another study in rural Tanzania [50] and Ethiopia [51]. Qualitative research from a similar study in rural Southern of Tanzania showed that mothers stated reasons for not frequently seeking PNC services including negligence and unplanned pregnancy and overall lack of knowledge of danger signs of newborn illness [20]. Our findings also showed that delivering at the health facility had a negative effect on the frequency of PNC, results confirmed by another study from the same region of Tanzania (Morogoro) [26]. However, our results with interaction terms showed that when ANC visit is low, the place of delivery had no statistically significant effect on PNC visit. However, the high attendance to ANC services had a positive and significant effect on number of PNC visit. That indicates that the facility delivery represents missed opportunity to promote PNC cares benefit for mothers and their newborns. This finding suggests that there is a need to improve the quality of maternal health care services during childbirth (counseling or health education and promotion) [52].

Our findings showed that sick newborns were more likely to be cared for than healthy newborns. Research from rural women in Nepal showed that health problems were significantly associated with having PNC [53]. That care was sough because of the health status of mothers and their newborns. Then, PNC is not functioning as prevention care. It is rather a parental response to illness during the postnatal period. Least healthy newborns would be receiving the most intensive care.

Our findings showed that the district of residence remains a predictor of service utilization. The low rate among mothers from Ulanga compared to those from Rufiji and Kilombero districts could be associated with the higher physical access to health facilities and lower density of health facilities in Ulanga compared to Rufiji and Kilombero. Compared to previous studies, which have demonstrated that a higher physical accessibility may increase maternal services utilization in Tanzania [50], Ghana [54] and Nepal [55], our results have not shown strong evidence that mothers who lived near to a health facility attended more frequently PNC services as compared to those who lived far from a health facility.

Consistent with prior empirical studies [56], the age of the mother had an independent influence on PNC utilization. This fact could be related to the risk factors associated with young mothers. Ethnicity and to a lesser extant, religion had a independent influence on PNC utilization in the study area as shown also in previous studies [57]. This could be explained by the underlying cultural beliefs and values systems of different ethnic groups, which influence health perceptions, needs, and behavior of their group members. Our study does provide the relative importance of ANC attendance in facilitating or hindering the utilization of PNC services. However, one review of ten studies that used similar multivariate analysis found that it was not possible to assess whether one specific factor was more important that the others in determining utilization [58]. The authors concluded that to be most effective, PNC interventions should address all barriers to utilization comprehensively and not target single barriers in isolation.

Overall, our study highlights the need for further research and program efforts aimed at increasing utilization of PNC services during this period of high risk for mothers and their newborns. In particular, there is a need to explore effective means of promoting routine preventive care PNC. Further research into utilization of PNC should combine individual level behavior data with child survival and neonatal mortality data to examine the link between health seeking behavior and health outcomes.

### Potential study limitations

The “real” distance to facility or time to reach the facility was not available. As such, it was not possible to assess with accuracy how the distance and the time-travel from household to closest health facility affected the PNC attendance. The household wealth was defined with only water and sanitation factors. Unfortunately, other household assets such as house roofing material, wall material, floor material etc. that are conventionally included in determining household wealth status using PCA method were not available.

Since endogeneity could arise from the possibility that PNC can be both a response to neonatal illness and a parental response to the need for preventive care, analyses control for maternal recall of illness so that RRR represent a net parental response to preventive care, adjusted for the potentially confounding effect of illness.

## Conclusion and policy implications

Despite the remarkable success in reducing child mortality [59], Tanzania has made no significant progress in reducing newborn deaths. An understanding of the factors that influence utilization of PNC by mothers and their newborns is helpful in identifying possible reasons for use and non-use. While the results of this study illuminate such reasons, further research is needed to contextualize the influence of these multi-level factors on individual utilization of PNC. To achieve the WHO recommended number of PNC in rural Tanzania, our findings suggest the need to provide PNC through the community-based primary health care and to improve the quality of maternal health services (counseling and education), during antennal and postnatal periods. Such an understanding can inform the Ministry of Health and Social Welfare in Tanzania in the formulation of national and local policies and programs to increase PNC utilization rates, with the ultimate goal of reducing preventable deaths taking place in the high risk postnatal period and beyond.
